# A slope method for the determination of electron energy for quality assurance

**DOI:** 10.1002/acm2.14369

**Published:** 2024-04-29

**Authors:** Larry W. Hill, David Jack

**Affiliations:** ^1^ Department of Radiation Therapy Genesis Care/21st Century Oncology, Fort Walton Beach Florida USA

**Keywords:** electron beam energy check, ionization, monthly quality assurance, TG‐141, TG‐198

## Abstract

**Background:**

Particle accelerators, manufactured for delivering patient radiation treatment, require numerous and frequent quality assurance measures. One of those is the periodic check for electron energy stability. The American Association of Physicists in Medicine has established requirements for this procedure. The current recommendation is to perform a ratio of two ionization points, one at *D*_max_ and another at a point approximately to the 50% depth, compared to a baseline as a relative check.

**Purpose:**

This ratio method is a sensitive measurement and sometimes produces results that are difficult to interpret or relate to acceptable tolerances. We sought to find a simple method that gives more stable results, which can be interpreted and related to energy changes.

**Method:**

We propose a method that takes two measurements on the descending portion of the shifted percent depth ionization (PDI) curves to calculate the slope, tangent to the *I*_50_ point, the point at which the ionization falls to 50% of its maximum value. We then used the slope measurement, compared to an established baseline, to relate energy.

**Results:**

After collecting data over a 3‐year period, we saw that standard deviations for the slope method have much less variability than the traditional ratio method. We were also able to correlate the slope results to ionization scans performed in water and found they were in better agreement than the traditional ratio method.

**Conclusion:**

The slope method does not require precise positioning since the slope remains relatively constant over the descending portion of the curve. Our data show that this results in an easier interpretative test of electron energy stability and delivers reliable feedback for quality assurance.

## 1 INTRODUCTION

This study provides an alternative method for determining relevant electron energy methodology, as it relates to quality assurance. Electron energy determination has been associated with the extrapolated range and/or, the depth of the 50% ionization/dose. The SCRAD protocol^1^ (1966) suggested several methods for determining electron energy that are time‐consuming, but the generally accepted test was the use of the extrapolated range. If the relative ionization in water was plotted as a function of depth, the practical range, *R*_p_, could be determined by extrapolating the descending portion of the curve to where it intersects the bremsstrahlung tail.

This point was then related to the energy by the Markus equation, *E*_0_ = (*R*_p_ + 0.376)/0.521 (cm), where *E*_0_ is the energy (MeV) at the surface of the phantom and *R*_p_ is the practical range of the electron beam. The Nordic Association of Clinical Physics^2^ updated this in 1980^3^ to *E*_p_,_0_ = 0.22+1.98*R*_p_*0.0025*R*_p_^2^ as an electron energy determination, where *E*_p,0_ is the most probable energy (MeV) at the surface. Subsequently, Task Group 21/25/51^4^, ^5^, ^6^ recommended using the 50% depth of the dose ‐depth curve, in the equation *E*_0_ = 2.33 * *R*_50_, for obtaining the mean incident energy. The more recent TG 70^7^ supplement to TG 25 recommends using *E*_0_ = 0.656 + 2.059* *R*_50_ + 0.022*R_50_^2^, where *R*_50_ is the depth in cm of the 50% dose.

The SCRAD protocol suggested using an ionization ratio (fixed depth measurement ratio to maximum ionization depth measurement) compared to a baseline as a more convenient method of checking energy stability on a regular QA schedule. Other AAPM reports (13)^8^ did not elaborate on specific testing details. Task Group 40^9^ provided more insight as to frequency (monthly) and tolerances of the energy checks. TG 142^10^ recommends 2%/2 mm for electron energy constancy checks on a monthly frequency. The checks specify 2% tolerance, but baseline ratio percentage changes do not translate directly into physical movement on the PDI curve and the 2 mm tolerance is unclear, but we assume it would be relative to *I*_50_.

Meyer et al.^11^ reported that determination of the electron energy by measuring the ratio of readings at two different depths is highly sensitive and can show large percentage ratio changes. Changes in energy, positioning of probe depth, and choice of ratio depths, can often make it difficult to determine if the measurement error is acceptable or requires further analysis. Meyer and colleagues observed that a 6 MeV electron could show a 15% deviation in ratio but still could be within 2 mm, while an 18 MeV beam might only deviate by 6% for the same 2 mm tolerance. The dilemma of whether to do further testing involves the time‐consuming water scanning system setup, with frequent findings of no observable changes in beam energy—a question not easily resolved. TG 198^12^ updates were more specific at recommending the measurement of two applicator depth‐dose curves; however, this is more time consuming. The *R*_50_ values are to be compared to a baseline with a tolerance of ±1 mm.

Numerous papers have provided more detailed information regarding depths and procedures, such as the divided depth method,^13^ a rapid method,^14^ and the use of energy‐range relationships.^15^ Other authors^16^, ^17^, ^18^, ^19^, ^20^ provide methods of quick energy checks through detector arrays, computed radiography systems, or wedge devices, but these require equipment that is often expensive or otherwise not available. We propose to use measurements at two depths along the tangent to the *I*_50_ portion of the descending ionization curve to measure the slope and relate directly to the electron energy.

## 2 METHOD AND MATERIALS

**FIGURE 1:**
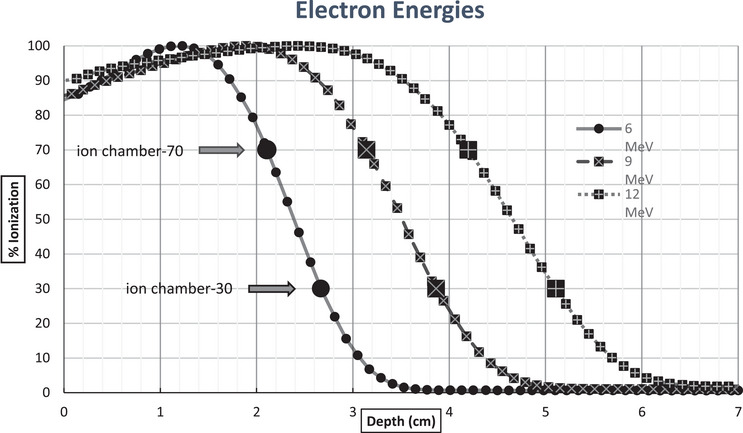
Chamber position for 70/30 slope.

Our institution initially required monthly TG‐51 dose outputs be done in water. Probe positioning can introduce error as I_50_ is on the steep descending portion of the curve and a small variation in probe placement can produce substantial differences. Our method of taking two measurements on the slope of the curve does not require as precise positioning since the slope remains relatively constant over the descending portion of the curve. The decision to pick two depths was centered on the observation that electron energy has been historically defined by *R*_50_ and/or the *R*_p_. By choosing two equidistant points from *R*_50_ (or *I*_50_) depth, we could produce a tangent line to the *R*_50_ (or *I*_50_) and a very reproducible slope value. This can also be related to the *R*_p_. It is evident that the electron energy slope changes with energy as shown in Figure 1. Increasing energy produces a shallower slope.

Our method was to choose two depths from the original commissioning scans, reflecting the ionization values of 70% and 30%. See Figure 1. We chose to use ionization data to simplify the process rather than converting it to dose. The commissioning scans were done with the IBA Blue Compact phantom 2D tank system with an IBA cc‐13 scanning ion chamber. The system positioning accuracy is quoted at ± 0.1 mm with the same position reproducibility. System leakage is quoted at < 200 fA (usually < 20 fA). The IBA cc‐13 is a small chamber with an active volume of 0.13 cc and a specified sensitivity of 3.6 nC/Gy and typical leakage of 3 fA.

When choosing measurement points, we decided to use depths that correspond to the effective point of measurement of the ion chamber (since our scanning software automatically shifts the data upward by 0.5*radius of the chamber). The slopes were calculated from the linear, descending portion of the commissioning scans.

Rather than set up our scanning system each month, we chose to use our TG‐51 ion chamber (which we use to determine dose checks at our reference depth) to establish a monthly QA check for electron energy. We then measure the two depths at the 70/30 points, normalized to the reference depth (which is on a relatively flat portion of the curve and usually at the maximum reading). This is done to give us a percentage value which is then used to determine the slope using the equation:

(1) $$\begin{array}{c} M=\left(\mathrm{Ion}70\%-\mathrm{Ion}30\%\right)/\left(d2-d1\right)\;\;\;\left(\%/cm\right) \end{array}$$

where Ion70% and Ion30% are the values at the 70% and 30% points, and d2 and d1 are the corresponding depths (cm) to the 70% and 30% points.

Equation (1) then gives us the baseline slope for our monthly QA checks. These values were measured with an Exradin A12 ion chamber connected to a Standard Imaging Premier 3000 electrometer. The A12 chamber has an active volume of 0.64 cc and an approximate sensitivity of 20 nC/Gy. Leakage was determined to be 2.5 fA.

Once we had a reference slope, for each energy, we compared the monthly QA measurement to the reference slope to determine changes.

We present three years of data. The data were taken from two Elekta Versa HD accelerators and one older (+10 years) Elekta Synergy accelerator, all with Agility MLC heads. Data were taken for four electron energies: 6, 9, 12, and 15 MeV.

The uncertainties of the linear accelerator (linac) system were not specifically addressed in this study. Rather, the commissioning and subsequent annual data were compared to the Elekta standard data. The electron energies from the three linacs of this study were not dosimetrically equivalent but matched well with the standard data sets. In addition, besides our slope method being relative measurements, it does not introduce any new uncertainties over the traditional, two depth method of periodically checking electron energy changes.

The errors and uncertainties that we did address were mechanical positioning of the ion chamber, the position reproducibility, pre‐irradiation leakage, and reproducibility of the measurements.

The initial positioning of the ion chamber was done by mirroring the chamber reflection at the water surface and verifying with a calibrated ruler at depth. The water tank used for the monthly QA with the A12 chamber was manufactured by Med‐Tec along with a 1D mechanical stepper with a scale of 0.01 mm. However, we estimated the position uncertainty to be approximately ± 0.2 mm. The reproducibility of positioning was estimated to be ± 0.2 mm. Overall position uncertainty being ± 0.3 mm.

Pre‐irradiation leakage was measured at 2.5 fA. Reproducibility of repeat measurements was less than 1% (usually < 0.5%) for all systems used in this study.

## 3 RESULTS

The results are presented for one linac only due to the amount of data. The slope baselines for the different linacs were similar yet still different, as expected since the electron energies were not dosimetrically matched. For future study, we hope to match our electron energies and explore the slope baselines for similarities. Presently, we recommend establishing specific baselines for each linac. We did explore data from other vendor linacs and saw similar results but a comprehensive study across multiple institutions is beyond the scope of this paper. The raw data was not smoothed or manipulated but only analyzed for statistical outliers using *Z* scores. The data was consistently within 2 standard deviations of the mean. All the lower electron energy data fit a normal distribution and remained reasonably stable (< 2%) over the measurement period. However, our 15 MeV energy did not remain stable after the first 2 years but began an energy drift trend during the third year.

**FIGURE 2:**
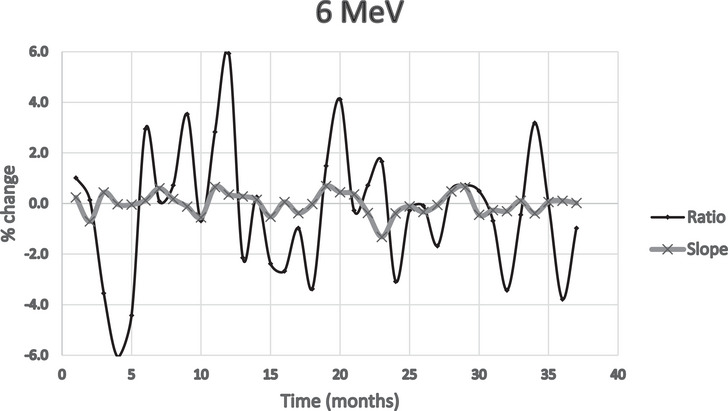
Percent change from baseline comparing ratio/slope methods (6 MeV).

**FIGURE 3:**
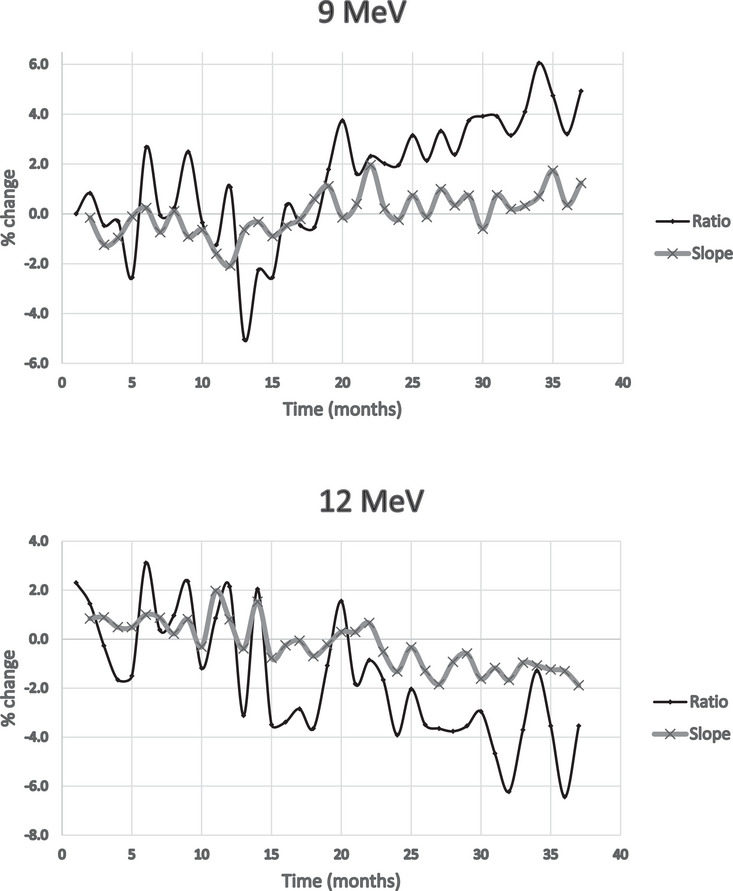
Percent change from baseline comparing ratio/slope methods (9 and 12 MeV).

**FIGURE 4:**
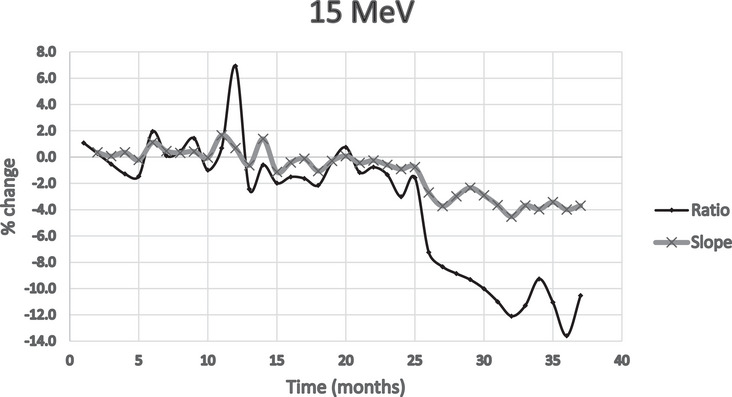
Percent change from baseline comparing ratio/slope methods (15 MeV).

Figures 2, 3, 4 show the percent deviation from the baseline value for both the slope and the traditional ratio method, which we define as the ratio of two ionization measurements, at *I*_dmax_ and approximately *I*_50_ depths. The slope numbers were stable over the period measured; only two measurements over 2%; excluding 15 MeV. The ratio method showed nine measurements exceeding 4%: again, excluding 15 MeV.

The issue, with the *I*_max_/*I*_50_ ionization ratio method, is determining what to do with values that exceed 2%, especially those exceeding higher values. The question was whether this reflected a real energy change that needed additional analysis or adjustment.

### 3.1 Comparative results

With the exclusion of the 15 MeV data of the last 11 months, the standard deviations were calculated for the percentage deviations from the reference, for all energies, and for both slope and ratio methods. See Table 1.

**TABLE 1:** Comparison of slope versus ratio standard deviations.

Nominal energy (MeV)	Slope	Ratio
6	0.44	2.57
9	0.87	2.32
12	0.97	2.62
15	0.73	1.92

A comparison of the two methods shows that the standard deviations for the slope method show less data spread than the traditional ratio method. The ratio method also had much higher deviations from the reference, with several months differing by over 4% versus 2% for the slope method.

The 15 MeV energy drift gave us an excellent opportunity to test and compare the different energy measurement methods. The initial measured slope values, for 15 MeV, over the first 2 years did not show changes greater than 2%, but within the last 11 months, relative slope values indicated an average drop of −3.8%, especially during the last 4‐month period, a deviation from the overall average.

**FIGURE 5:**
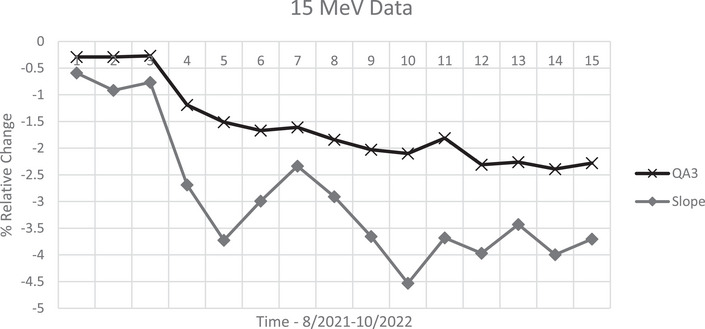
15 MeV % relative change—daily QA3 to slope values.

We analyzed data from our Daily QA3 device, over the same period for 15 MeV and compared the results to slope data. The relative baseline change followed the same trend as our slope measurements. During the same period, the Daily QA3 showed a drop in energy of 2–2.4%, while the slope change was on the order of 3.5–4%. See Figure 5.

**FIGURE 6:**
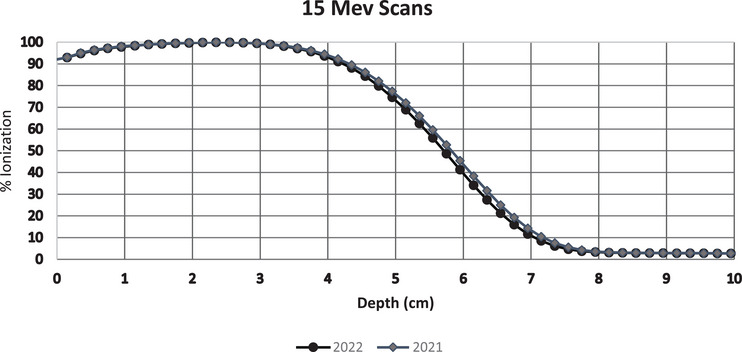
Annual water scan curves 15 MeV—2021 versus 2022.

We summarized our results with water tank ionization scans of the 15 MeV and compared them to the annual scan done the prior year. We found a 1 mm shift in the descending portion of the ionization curve, which aligns well with what we were seeing in the slope indicators. IBA software E_0_ calculations agree, showing 13.7 MeV for 2021 scans, with 13.5 MeV for 2022, indicating a relative decrease of 1.5%. See Figure 6.

We observed a correlation between the plotted slope values versus measured energies (from our water scanning software), as defined by 2.33* R_50_. This plot produces an exponential equation:

(2) $$\mathrm{Energy}=32.311*e^{\left(0.0247*\mathrm{slope}\right)}$$

We initially used Equation (2) to compute relative energy changes and how the various methods of checking electron energy compared. See Table 2.

**TABLE 2:** Relative baseline change method comparison.

	15 MeV % baseline changes				
	Slope	Ratio	QA 3	Equation 2	Scans
**%** Change	−3.8%	−11%	−2.4%	−3.1%	−1.5%

*Note*: The traditional ionization ratio indicated an average change of 11%, which did not correlate as well with the other methods [slope measurements, QA3, Equation (2), and annual water scans].

**FIGURE 7:**
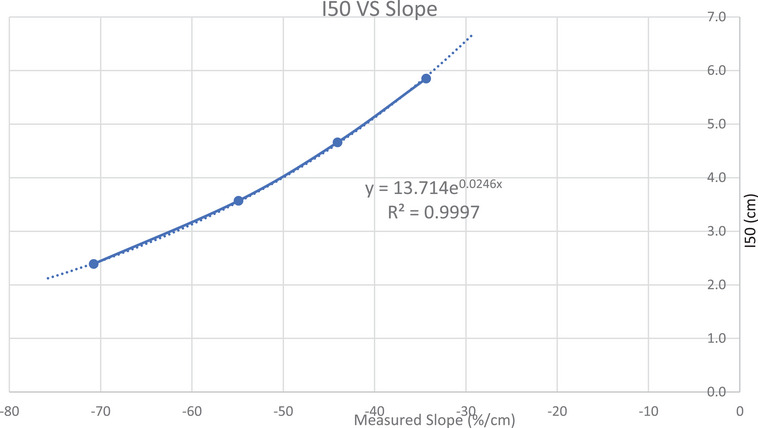
Plot of *I*_50_ versus measure slope data.

However, using Equation (3) still left us with the same dilemma of trying to understand the significance of relative percentage changes. By plotting *I*_50_ values versus measure slope data, as shown in Figure 7, we observed a similar relationship, Equation (3), to *I*_50_.

An exponential trend analysis produces Equation (3):

(3) $$I_{50}=13.714e^{\left(0.0246*\mathrm{slope}\right)}$$

Equation (3) gives a more practical result that can be used to determine the need for energy adjustment, as it produces a result in distance shift of I_50_.

The slope method enabled us to convert slope values into I_50_ shifts, using Equation (3). We were then able to establish recommendations for tolerance standards. By predicting I_50_ shifts of ± 1 and ± 2 mm, we saw a common relative slope value change of approximately 2% and 4%, respectively. See Table 3.

**TABLE 3:** Percent change in slope relative to *I*_50_ shifts in mm.

% Slope change to *I*_50_ shift		
Energy	± 1 mm	± 2 mm
6	2.4	4.8
9	2.1	4.2
12	2.0	4.0
15	2.0	4.0

Equation (3) was derived from the IBA commissioning data for the linac presented in this paper. Since there are differences in energies across facilities, Equation (3) differs as well for the other two linacs. Z tests show statistical differences between each equation but not surprisingly, since they are not matched energies. Equation (3) for this data (and the other linacs) have a very good coefficient of determination fit (*R* ^2^ = 0.9997 – see Figure 6), which suggests the model is a good predictor of I_50_. All the different equations have coefficients great that 0.997.

The monthly slope QA measurements were used solely for baseline checks while the IBA slopes were used to establish the depths for 70%/30% points and to derive Equation (3) for evaluation of I_50_ movement.

## 4 CONCLUSION

From our results [Equation (3) and Table 3], we set our warning tolerance to a 2% change in slope measurement that reflects an approximate 1 mm shift and a 4% change as the limit, reflecting an approximate 2 mm shift in the electron curve at *I*_50_. The slope method of analyzing electron beam energy constancy is a quick and inexpensive way to check for changes on a monthly frequency, allowing easy and meaningful assessment for quality control of electron energy. Comparison of the slope method to the traditional ionization ratio method showed the extreme sensitivity of the ratio method and the difficulty of determining actions, while the slope method is more consistent and meaningful.

Initial investigation suggests that each linac would require establishing slope baseline values and should be independent of the type of ion chamber used to measure the slope points. I would encourage future studies to determine if matching energies would be helpful in unifying Equation (3) and to see what differences there may be between different models and manufacturers of accelerators.

In conclusion, we feel that the slope method offers an alternative means of obtaining useful action levels for the measurement of electron energy, with only one additional measurement point per energy.

## AUTHOR CONTRIBUTIONS

L. Hill conceived of the presented idea, developed the theory, and collected the data. All computations were done by L. Hill. L. HIll and D. Jack verified the analytical methods and discussed the results and final manuscript.

## CONFLICT OF INTEREST STATEMENT

The authors have no conflicts of interest to disclose.
